# The Sialic Acid-Dependent Nematocyst Discharge Process in Relation to Its Physical-Chemical Properties Is a Role Model for Nanomedical Diagnostic and Therapeutic Tools

**DOI:** 10.3390/md17080469

**Published:** 2019-08-12

**Authors:** Ruiyan Zhang, Li Jin, Ning Zhang, Athanasios K. Petridis, Thomas Eckert, Georgios Scheiner-Bobis, Martin Bergmann, Axel Scheidig, Roland Schauer, Mingdi Yan, Samurdhi A. Wijesundera, Bengt Nordén, Barun K. Chatterjee, Hans-Christian Siebert

**Affiliations:** 1 Institute of BioPharmaceutical Research, Liaocheng University, Liaocheng 252059, China; 2 RI-B-NT—Research Institute of Bioinformatics and Nanotechnology, Schauenburgerstr. 116, 24118 Kiel, Germany; 3 Neurochirurgische Klinik, Universität Düsseldorf, Geb. 11.54, Moorenstraße 5, 40255 Düsseldorf, Germany; 4 Institut für Veterinärphysiolgie und-Biochemie, Fachbereich Veterinärmedizin, Justus-Liebig-Universität Gießen, Frankfurter Str. 100, 35392 Gießen, Germany; 5 Department of Chemistry and Biology, University of Applied Sciences Fresenius, Limburger Str. 2, 65510 Idstein, Germany; 6 RISCC—Research Institute for Scientific Computing and Consulting, Ludwig-Schunk-Str. 15, 35452 Heuchelheim, Germany; 7 Institut für Veterinäranatomie, Histologie und Embryologie, Fachbereich Veterinärmedizin, Justus-Liebig-Universität Gießen, Frankfurter Str. 98, 35392 Giessen, Germany; 8 Zoologisches Institut-Strukturbiologie, Zentrum für Biochemie und Molekularbiologie, Christian-Albrechts-Universität, Am Botanischen Garten 19, 24118 Kiel, Germany; 9 Biochemisches Institut, Christian-Albrechts Universität Kiel, Olshausenstrasse 40, 24098 Kiel, Germany; 10 Department of Chemistry, University of Massachusetts Lowell, 1 University Avenue, Lowell, MA 01854, USA; 11 Department of Chemical and Biological Engineering, Chalmers University of Technology, SE-41296 Gothenburg, Sweden; 12 Department of Physics, Bose Institute, 93/1, A P C Road, Kolkata 700009, India

**Keywords:** nematocyst discharge process, theoretical model, polysialic acid (polySia), nematogalectin, nanomedical devices

## Abstract

Formulas derived from theoretical physics provide important insights about the nematocyst discharge process of Cnidaria (Hydra, jellyfishes, box-jellyfishes and sea-anemones). Our model description of the fastest process in living nature raises and answers questions related to the material properties of the cell- and tubule-walls of nematocysts including their polysialic acid (polySia) dependent target function. Since a number of tumor-cells, especially brain-tumor cells such as neuroblastoma tissues carry the polysaccharide chain polySia in similar concentration as fish eggs or fish skin, it makes sense to use these findings for new diagnostic and therapeutic approaches in the field of nanomedicine. Therefore, the nematocyst discharge process can be considered as a bionic blue-print for future nanomedical devices in cancer diagnostics and therapies. This approach is promising because the physical background of this process can be described in a sufficient way with formulas presented here. Additionally, we discuss biophysical and biochemical experiments which will allow us to define proper boundary conditions in order to support our theoretical model approach. PolySia glycans occur in a similar density on malignant tumor cells than on the cell surfaces of Cnidarian predators and preys. The knowledge of the polySia-dependent initiation of the nematocyst discharge process in an intact nematocyte is an essential prerequisite regarding the further development of target-directed nanomedical devices for diagnostic and therapeutic purposes. The theoretical description as well as the computationally and experimentally derived results about the biophysical and biochemical parameters can contribute to a proper design of anti-tumor drug ejecting vessels which use a stylet-tubule system. Especially, the role of nematogalectins is of interest because these bridging proteins contribute as well as special collagen fibers to the elastic band properties. The basic concepts of the nematocyst discharge process inside the tubule cell walls of nematocysts were studied in jellyfishes and in Hydra which are ideal model organisms. Hydra has already been chosen by Alan Turing in order to figure out how the chemical basis of morphogenesis can be described in a fundamental way. This encouraged us to discuss the action of nematocysts in relation to morphological aspects and material requirements. Using these insights, it is now possible to discuss natural and artificial nematocyst-like vessels with optimized properties for a diagnostic and therapeutic use, e.g., in neurooncology. We show here that crucial physical parameters such as pressure thresholds and elasticity properties during the nematocyst discharge process can be described in a consistent and satisfactory way with an impact on the construction of new nanomedical devices.

## 1. Introduction

The structural development of Hydras can be discussed under the general aspects of morphology generation as it has been published by Alan Turing in his pioneering paper already in the year 1952 [1]. Hydra is an ideal organism to study morphogenetic models directly derived from the laws of mathematics, theoretical physics and biophysical chemistry. We focus on different Cnidaria species as model organisms in general because their biological principles, especially the nematocyst discharge processes are not too complex for descriptions which originate from mathematics and theoretical physics. This is therefore a proper example which shows that complex processes in living nature can be described with such methods [1,2,3,4,5,6,7,8,9]. These model descriptions have to be combined with data from structural biology and biochemistry. The nematocyst capsule is filled with γ-glutamate polymers, which binds a 2 M concentration of cations, thereby generating a high osmotic internal pressure of more than 15 MPa [10,11]. A sialic acid dependent chemical-mechanic trigger initiates exocytosis in a living model organism [12,13]. 

The nematocyst ejects a stylet with a highly elastic tubule under the pressure of nearly 7 GPa and a velocity of over 15 m/s [14]. This punches a hole into the prey’s integument and toxins are injected. In this case, one can identify nearly identical surface signatures on the cells of preys or predators and on malignant tumor cells a nematocyst-like device could be the bionic blue-print of a new nanomedical approach in cancer diagnostic and therapy. Since the nematocyst has a mechanic-chemical receptor on its surface which interacts with polysialic acid (polySia) fragments and protein-like contact-molecules in a specific way, the corresponding glycan moieties are proper candidates. PolySia occurs on the cell surfaces of cnidarians preys and predators as well as on many tumor cells. We have chosen nematocysts from Hydra and tissue materials from certain jellyfishes in order to start our theoretical and experimental analysis. Hydra and other cnidarian organisms have proven that these organisms are feasible objects to study biological phenomena such as immunological principles and stem cell maintenance [15,16,17,18,19,20,21]. Carbohydrate-protein interactions play an important role in interaction processes on cell surfaces [22,23,24,25,26,27,28,29]. When the nematocyst discharge process is studied on a molecular level, carbohydrate-protein interactions are of central importance. It has been proven by Alan Turing on a fundamental morphological level that differential equations describe the time-dependence of activator and inhibitor concentrations in Hydra. Such equations can be solved and used for computational simulations which are in accordance with the Gierer-Meinhardt model [3,4,5,30]. The nematocyst discharge process itself is often characterized as a kind of Coulomb explosion which has recently been discussed for a different phenomenon in anorganic chemistry, analytic chemistry and material sciences [31,32,33]. We argue here that the central effect of the nematocyst discharge process, the expulsion of the stylet is only loosely related to the Coulomb explosions described in the literature cited above. The nematocyst discharge process is initiated by Coulombic stress due to the buildup of charge inside the nematocyst and at some threshold values of this stress the stylet is ejected explosively (suddenly and with large acceleration). It is therefore obvious that the nematocyst discharge process should be described successfully with formulas from theoretical physics. Further insights concerning the boundary conditions of the discharge process in respect to material requirements and surrounding conditions are provided by biophysical experiments such as QCM (Quartz Crystal Microbalance) methods, EM - Electron Microscopy (especially TEM—Transmission Electron Microscopy), AFM—Atomic Force Microscopy, SPR—Surface Plasmon Resonance, MS—Mass Spectrometry and NMR—Nuclear Magnetic Resonance techniques. Furthermore, X-ray crystallographic structure models from the protein data bank are essential to construct nematogalectin architectures which show the bridging activity of these molecules with proteoglycan fragments in the tubule cell wall. The nano-architecture in which mini-collagens, proteoglycans and nematogalectins are involved is responsible for the physical properties of the nematocyst membrane and the tubule system to which the stylet is connected [21]. 

The main components of the capsule wall of nematocysts, the explosive organelles of Hydra, jellyfish, corals and other Cnidaria, are collagens which are termed mini-collagens because of their shortest known collagen-like sequence of Gly-Xaa-Yaa repeats. Mini-collagens are the major components of the Hydra nematocysts capsule wall where they form a tight three-dimensional network by disulfide reassembling of their terminal cysteine-rich domains. Mini-collagens contain a short collagen triple helix flanked by polyproline stretches and terminal cysteine-rich domains. These N- and C-terminal proline- and cystine-rich domains of mini-collagen-1 are relatively short peptides (23 and 24 residues, respectively) containing 6 cysteine residues in an identical sequence pattern. The related synthetic peptides were found to refold in an oxidation process almost quantitatively into one topoisomer. Surprisingly, the two refolded domains exhibit different cystine frameworks despite their identical cysteine pattern and a high sequence homology. Therefore, the structural elements responsible for their distinct structures were analyzed to gain possible information about the mechanisms of mini-collagen disulfide rearrangement in their assembly into polymeric fibers. With better information on this process, mini-collagens containing functional domains of type I and IV collagens could be used for the preparation of mechanically highly resistant frameworks for cell adhesion and thus constitute promising innovative biomaterials [34]. However, their architecture is also of interest when nematocyts in various intact nematocytes are tested concerning its diagnostic feasibility when coming into contact with sialic acid coated surfaces. It is possible to reference the corresponding molecular concentration and density with tailor-made sialic acid-coated nanoparticles as outlined later. Sialic acids trigger the initiation of the Coulomb repulsion inside the nematocyst in an intact nematocyte. In this initiation process sialic acid mediated protein–carbohydrate interactions on the surfaces of nematocysts play a crucial role. It is our aim to figure out whether such bionic concepts provide guidelines for nanomedical and nanopharmaceutical devices in order to improve cancer diagnostic and therapies. The chemical and mechanical properties of the nanomedical tools from cnidarian origin can be studied in an ideal way when using first the well described Hydra nematocysts as blueprints. We show here that classical physics in combination with structural biology and theoretic biochemistry [1,2,7,8,9,24,35,36,37,38,39,40,41] is essential for an accelerated biophysical development of new diagnostic strategies analyzing the spread of highly-malignant tumor cells. It has to be emphasized here that this is only possible with in intact nematocytes. The corresponding vessels in a living nematocyte must have a robust consistence which enable them to resist the forces of a nematocyst discharge process. As outlined by ’t Hooft [9]: “Instruments of atomic dimensions could be used for any purpose imaginable, but particularly in medical sciences. Remote controlled robots could be sent to any part of the human body to resolve problems. Less advanced, passive robots could be attached to capsules containing medications; the capsules would be injected into the body, with the robots ensuring administration of the medication in the right dosages for weeks or even years.” Our approach discussed here emphasizes that blueprints of bionic robot systems already exist in form of Cnidaria nematocysts which can be used due to their molecular interaction specificity, as nanomedical tools. This is extremely promising because of their polySia-directed target function as diagnostic and therapeutic devices in order to identify tumor tissues, especially, polysialic acid rich cancer cells coated in a highly characteristic way which often remain in the tumor-hole after a neurosurgical intervention due to its location in close proximity to crucial regions of the brain.

## 2. Results

**Figure 1:**
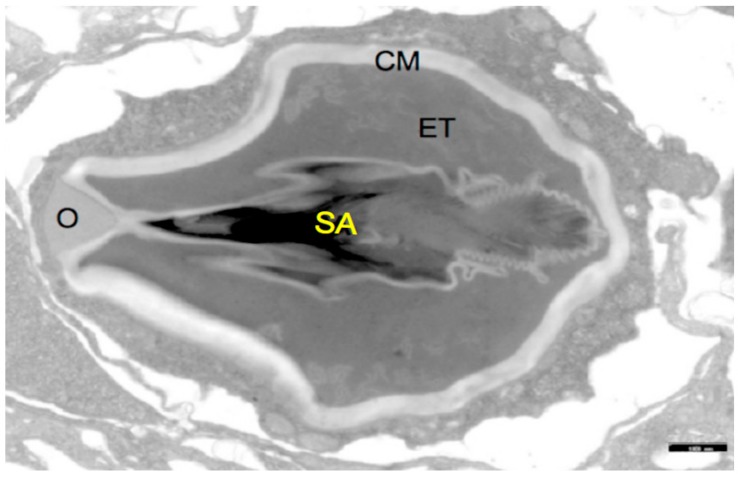
Structure of a Hydra nematocyst: Stylet apparatus with large and small spines (SA), external tubule (ET), capsule membrane (CM), operculum (O). The black scale bar in the lower right corner corresponds to 1 μm.

**Figure 2:**
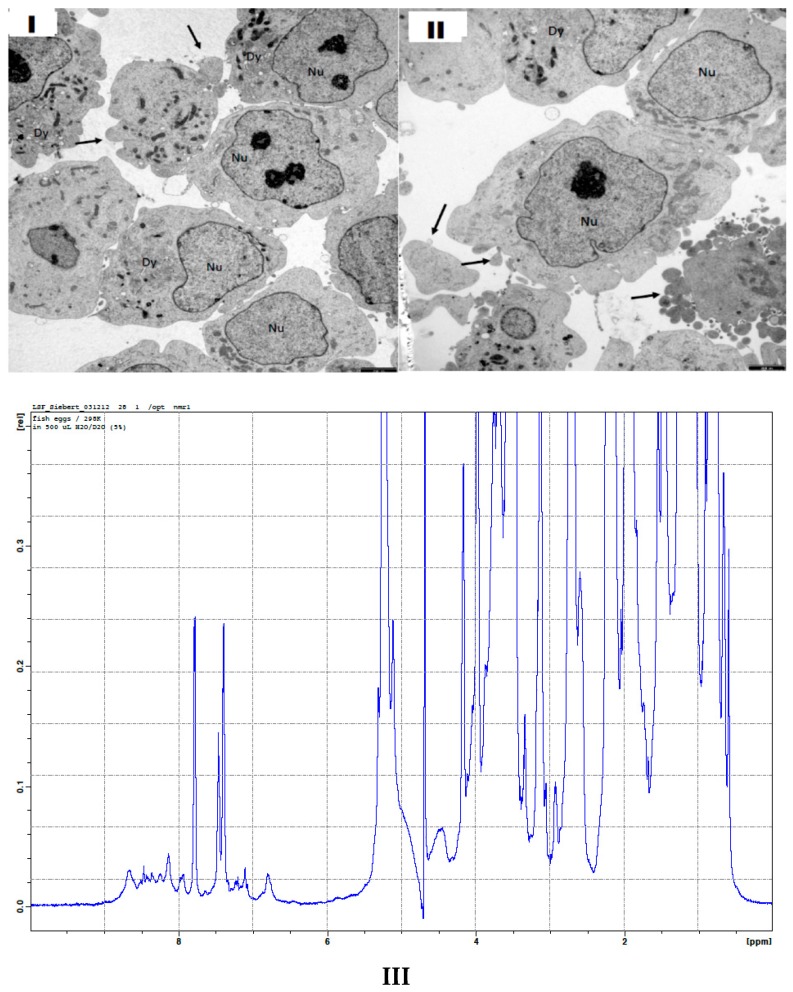
Structures of (**I**,**II**) neuroblastoma cells from a cell-line as revealed by transmission electron microscopy (TEM). Neuroblastoma cells show differences in their plasticity which could be of importance for an attack with a nanomedical device. (**III**) 1D proton NMR spectrum of broken fish eggs after their destruction in a nematocyst discharge process. A comparison with a 1D proton NMR spectrum of intact fish eggs is shown in Supplementary Materials.

The morphologies and consistencies of the drug-containing vessels (Figure 1) and their potential targets (Figure 2) were analyzed in detail using electron microscopy (EM), especially, transmission electron microscopy. The structural parameters obtained from Figure 1 are essential to define the boundary conditions of our theoretical model which is described below. When analyzing potential target structures for the drug-containing vessels, it turned out that malignant neuroblasts from a neuroblastoma cell line show peculiar bubble-like morphologies in the electron microscopical pictures (see arrows in Figure 2). This argues in favor of an alteration in the surface consistency of the corresponding cell membranes and the existence of peculiar topologies of special target regions. Besides the occurrence of polySia moieties on the tumor cells, such topological and morphological data are of highest importance to identify the Achilles heel of cancer cells. Furthermore, an extremely robust but also elastic consistency of the targeting cells is essential for our nanomedical devices. Under special consideration are here the nematocyte wall and the stylet-connected tubule system. The stylets of the nematocysts are ejected by a specific biological signal (sialic acids, especially polySia) which is present on fish eggs but also on various tumor cells [42,43]. The consistence of the collagen strands and of the nematogalectins which occur in the tubule walls [44,45] have to be studied on a sub-molecular level. This is also the case for the proteoglycans [36] in the nematocyst’s cell wall and the collagens in the exumbrella tissue of jellyfishes after their extraction. It turned out that biophysical methods which are based on QCM (Quartz Crystal Microbalance) techniques are feasible and helpful tools (Table 1) for testing the state of the extracted collagen-strands of jellyfish exumbrella tissues (Table 1).

A theoretical model which is discussed under Section 2.1 can be used to analyze experimental data in a more sophisticated way. As documented in Figure 1; Figure 2 nematocyst cells from Hydra and brain-tumor cells with a high concentration of polySia molecules on their surface as well as fish-eggs with a comparable polySia density are available for complementary biophysical and biochemical experiments. The theoretical model provides a clear standard description in which certain physical parameters are addressed. NMR experiments in which fish-eggs are used [46] as suited model targets, e.g., (Figure 2III), can be designed in a better way when the theoretical model description is applied in a suited way. Furthermore, the influence of certain ions on the nematocyst discharge process can be studied in the framework of certain NMR experiments in order to shed light on questions which are related to neurological diseases and mental disorders [47]. In full agreement with Alan Turing’s vision, it is indeed possible to use the partial simplicity of cnidarian organisms to answer basic questions with tools from applied mathematics and theoretical physics.

### 2.1. Theoretical Model 

We describe the discharge of the nematocyst as a five-stage process. The first stage comprises the triggering of the cnidocil, where an external chemical-mechanical signal provides the key-signal for the Coulomb explosion. After the process has been started by the external signal, it is translated to an internal Ca^2+^ signal initiating the next stage of the discharge process. In the second stage, a chemical trigger initiates proteolysis in the poly-*γ*-glutamic acid (PGGA) resulting in an increase of the H^+^ concentration in the nematocyst. Since the diffusion constant of H^+^ in water is large, ambipolar diffusion of H^+^ through the semipermeable wall of the nematocyst leaves behind a negatively charged matrix. The electrostatic potential developed between the centre of the nematocyst and the surface, is termed the Donnan potential. The charge density distribution in the nematocyst would be ideally governed by the expression:

(1) $$\nabla^{2}\ln\left(1+\frac{\rho }{en_{0}}\right)=\frac{\mu \rho }{\varepsilon D}$$

where $n_{0}$ is the initial concentration of H^+^ ions, *μ* is the mobility and *D* is the diffusion constant of H^+^ ions through the membrane and ε is the dielectric constant.

In the third stage, the charge density of the negatively charged matrix would give rise to a high pressure which would be governed by

(2) $$p=-\frac{\partial U}{\partial V}$$

where *V* is the volume of the nematocyst and *U* is the internal energy (electrostatic potential energy). The internal energy is given as:

(3) $$U=\frac{1}{2}\int \rho \left(\vec{r}\right)\int \frac{\rho \left(\vec{r^{\prime }}\right)}{4\pi \varepsilon \left|\vec{r}-\vec{r^{\prime }}\right|}{\mathrm{dV}}^{\prime }\mathrm{dV}$$

where ρ is the charge density. When the pressure exceeds a threshold value, the operculum opens up and after a second threshold the stylet, which is held back by a tough collagen bottle-neck, overcomes the restraint and is ejected at an immense speed. This stage could be termed as the Coulomb explosion stage as the movements take place at nanosecond time scales. Subsequently it gives rise to accelerations in the range of few million times that due to gravity and this explosive process is initiated by Coulomb repulsion, which is very much like a Coulomb explosion. 

In the fourth stage, the opercular chamber is distended by the proteolysis of the matrix in the opercular chamber and loss of H^+^ ion from it. This further pushes out the stylet thereby locking it with barbs in the victim or in some cases disposes off the outer sheath of the stylet. In the fifth stage, the tubule everts (i.e., turns inside out) through the hollow stylet and itself, exposing the poisonous barbs into its victim.

Here, assuming a homogeneous and spherical nematocyst we get

(4) $$\frac{1}{r^{2}}\frac{\partial }{\partial r}r^{2}\frac{\partial }{\partial r}\ln\left(1+\frac{\rho }{{\mathrm{en}}_{0}}\right)=\frac{\mu \rho }{\varepsilon D}$$

which can be scaled to a dimensionless form with $\lambda =\sqrt{\frac{{\mu \mathrm{en}}_{0}}{\varepsilon D}}$, x=λr and $y=\frac{\rho }{{\mathrm{en}}_{0}}=y\left(x\right)$, as,

(5) $$\frac{1}{x^{2}}\frac{d}{\mathrm{dx}}x^{2}\frac{d}{\mathrm{dx}}\ln\left(1+y\right)=y$$

Here $n_{0}$ is the initial number density of [H^+^], $\rho =-e\left(n_{0}-n\right)$ is the charge density, μ and *D* are the mobility and diffusion constant of [H^+^] through the semipermeable membrane, ε is the dielectric constant of water and e is the charge of the electron.

The boundary conditions y(∞)=0 and $y^{\prime }\left(\infty \right)=0$ does not aid in the solution of this nonlinear equation and does not reflect the spherical shape of the nematocyst. Since the charge buildup would be mostly near the inner surface of the nematocyst, a simple linear model is used, which would be valid near the surface u=R−r of the nematocyst and also assuming that $\rho \;{<<\mathrm{en}}_{0}$ one can write

(6) $$\frac{d^{2}}{{\mathrm{du}}^{2}}{\rho =\lambda }^{2}\rho$$

which can be solved to give $\rho =-{\mathrm{en}}_{0}\exp\left(-\lambda \left(R-r\right)\right){=\rho }_{0}\exp\left(-\lambda \left(R-r\right)\right)$, assuming that at *r* = *R* all H^+^ has diffused out.

This gives the Coulombic self-potential energy as *U* as

(7) $$U=\frac{2\pi \rho_{0}^{2}}{\lambda^{5}\varepsilon }\left\{\left(X^{3}-\frac{7}{2}X^{2}+\frac{11}{2}X-\frac{11}{4}\right)-4\left(X-1\right)\exp\left(-X\right)-\frac{5}{4}\exp\left(-2X\right)\right\}$$

where X=λR and a Donnan potential 

(8) $$\varphi =\frac{\rho_{0}}{\lambda^{2}\varepsilon }\left\{\left(X-1\right)+\exp\left(-X\right)\right\}$$

This gives rise to the Coulombic pressure ($p=-\frac{\partial U}{\partial V}$) which gives

(9) $$p=\frac{\rho_{0}^{2}}{2\varepsilon \lambda^{2}X^{2}}\left\{\begin{array}{c} -\left({3X}^{2}-7X+\frac{11}{2}+4(X-2)\exp(-X)+\frac{5}{2}\exp(-2X)\right)\\ +\frac{4(X-1+\exp\left(-X)\right)[(X^{3}-\frac{7}{2}X^{2}+\frac{11}{2}X-\frac{11}{4})-4\left(X-1\right)\exp\left(-X\right)-\frac{5}{4}\exp\left(-2X\right)]}{(X^{2}-2X+2-2\exp\left(-2X)\right)} \end{array}\right\}$$

One easily observes that in the limit λ→0 (a homogeneously charged sphere) this reduces to the usual

(10) $$p=\frac{\rho_{0}^{2}R^{2}}{15\varepsilon }$$

with a Donnan potential $\varphi =\frac{\rho_{0}R^{2}}{2\varepsilon }$, and, in the limit λ→∞ (a thin spherical shell of charge, with surface density ${\sigma =\rho }_{0}/\lambda$) this reduces to the usual

(11) $$p=\frac{\sigma^{2}}{2\varepsilon }$$

with a Donnan potential $\varphi =\frac{\sigma R}{\varepsilon }$.

The stylet emission is governed by the equation of a static balance of forces

(12) $$\pi \beta^{2}p={\left(-\frac{\partial \beta }{\partial \xi }\right)}_{\xi >\xi_{1}}\frac{2\pi \alpha Y(\beta -\beta_{0})}{\beta_{0}}$$

where the term on the left is the force on the stylet due to the Coulomb pressure, while the term on the right hand side is the restoring force on the stylet due to elastic materials (the elasticity of the nematocyst wall and the escape orifice is provided by minicollagen fibrils) restraining the emission of the stylet. The elastic material restraining the stylet is forced to be extended to the radius β(p) from the unstretched radius $\beta_{0}$ due to the pressure *p*. The radius (β) of the stylet is assumed to be a function of the axial position (ξ) and that the maximum radius of the stylet occurs at $\xi_{1}$. The Young’s modulus of the elastic material surrounding the stylet is *Y* and is of a cross section α.

**Figure 3:**
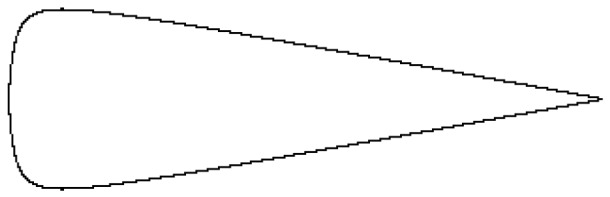
The shape of the stylet for a=0.2, l = 1 and d=0.01 as given by Equation (12), please see theoretical part.

Assuming that the shape of the stylus is governed by the equation

(13) $$\beta =\frac{a\xi \left(1-\xi \right)}{d+\xi }$$

where β is the radius at the axial position ξ, with slopes of al/d and −al/(d+l) at ξ=0 and *l*, and a maximum radius $\beta_{x}=a\left(l+2d-2\sqrt{d\left(l+d\right)}\right)$ at $\xi_{1}=\sqrt{d\left(l+d\right)}$. For certain values of *a*, *l* and *d*, it looks like a carrot (e.g., a=0.2, l = 1 and d=0.01) as in Figure 3. 

**Figure 4:**
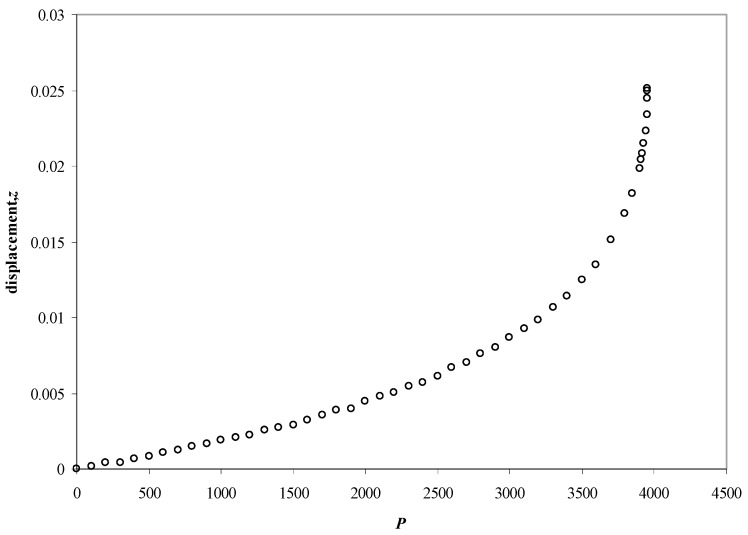
The displacement of the stylet (a = 0.2, l = 1, and d = 0.01) as a function of the applied pressure (in units of Y). The threshold pressure is about 3960 and all lengths are in units of the length of the stylet, please see theoretical part.

The displacement

(14) $$\delta =\frac{\beta \left(0\right)-\beta \left(P\right)}{2a}+\sqrt{{\left(\frac{l}{2}-\frac{\beta \left(P\right)}{2a}\right)}^{2}-\frac{d\beta \left(P\right)}{a}}-\sqrt{{\left(\frac{l}{2}-\frac{\beta \left(0\right)}{2a}\right)}^{2}-\frac{d\beta \left(0\right)}{a}}$$

increases nonlinearly with the pressure *P* (Figure 4), till at a certain threshold pressure *β*(*P*_thres_) = *β* the displacement diverges, and the stylet is ejected. The threshold pressure varies as

(15) $$P_{\mathrm{thres}}\approx \frac{\left(1-d\right)\mathrm{Ya}\alpha }{{2r}_{0}^{2}}$$

where *P*_thres_ and Y have the same dimensions, and all the lengths are scaled by the length *l* of the stylus.

The stylet can only accelerate after the pressure exceeds the threshold pressure and the acceleration of the stylus is then ${\pi R}^{2}p_{\mathrm{thres}}/m$. The maximum acceleration ($f_{\max}$) of the stylus will be given by

(16) $$f_{\max}=\frac{P_{\mathrm{thres}}{\pi R}_{x}^{2}}{m}$$

With $R_{x}$ is the maximum radius and *m* is the mass of the stylet. In terms of the parameters described above, one may represent this acceleration as,

(17) $$f_{\max}=\frac{Y\alpha \pi a^{3}(1-d)}{2(1.18+7d)mr_{0}^{2}}$$

and can be very large for small values of $r_{0}$. Here we see that very large accelerations are possible if the radius of the exit orifice ($r_{0}$) is small, and if the shape parameter (*a*) is large. The threshold pressure is proportional to the elastic modulus (*Y*) of the capsule and hence the exit orifice. Now these findings have to be correlated with experimental settings. In order to do this, biophysical, biochemical and cell-biological approaches have to be combined in a proper way. In this context it is of highest importance that *in silico* molecular modelling calculations support these approaches in an appropriate way. For a successful application of the theoretical model, it is of importance to know further details of the materials/molecules which constitute the corresponding parts in a cnidarian organism. This is outlined in the following paragraph. 

### 2.2. Biophysical Experiments 

**Figure 5:**
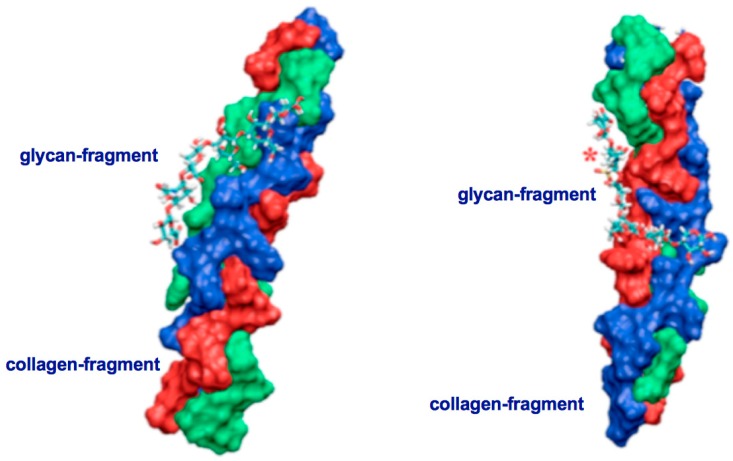
Triple-helical collagen strands in complex with non-sulfated (left side, orientation **a**) and sulfated (right side, orientation **b**) glycan-fragments. These models represent typical interaction state out of a great variety of other collagen- proteoglycan complexes. Sulfur-groups are highlighted by a red asterisk (*). Our results are in full agreement with AFM (Atomic Force Microscopy) and *in silico* studies on collagen strands interacting with proteoglycans [36].

**Figure 6:**
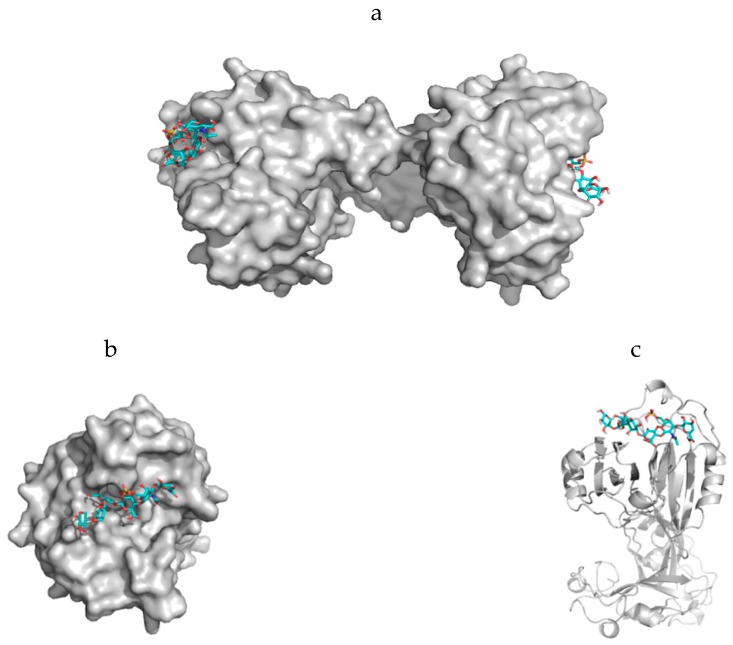
Model presentations of nematogalectin, a lectin which occurs in the capsule membrane (CM) of nematocysts. Interactions exist between parts from the carbohydrate chains of proteoglycans with collagen strands (Figure 5c) as well as highly specific interactions between certain moieties of the proteoglycan non-sulfated carbohydrate chains and the lectin are responsible for the cell membrane (CM) properties. Our calculations show that sulfated carbohydrates are also tolerated. The best template structure in the protein data bank is 2ZX2.pdb from the rhamnose binding lectin CSL3 [71]. (**a**) Surface presentation of the nematogalectin—showing its function as bridging molecule with two proteoglycan fragments in a stick structure. (**b**) Surface presentation—direct view on the binding pocket. (**c**) Ribbon structure of one part of the nematogalectin in complex with a proteoglycan fragment in a stick structure.

Two major things have to be taken into consideration before a nematocyst-like device can be used as nanomedical diagnostic tool for a target directed tumor cell-attack and as promising source for the design of innovative therapeutic tools in cancer therapy. Firstly, PolySia specific targeting by lectins, especially, mini-lectins which mimic the receptors on the nematocyst surface must be defined in detail [25,38,39,48]. Secondly, the interactions between the molecules in the nematocyst’s cell wall (mini-collagens as well as nematogalectins) and in its stylet-connected tubule system have to be described in detail on a sub-molecular level in a consistent way (Figure 5a,b and Figure 6a–c). Figure 5 shows two identical conformations at two different orientations. On the left side orientation a is shown and on the right side orientation b. For comparison, it is also of importance to analyze the collagen strands in the exumbrella tissue of jellyfishes with Quartz Crystal Microbalance (QCM) techniques. 

### 2.3. QCM Analysis of Collagen Fragments from Cnidaria 

It is essential to combine our theoretical results with data obtained by biophysical experiments. The correlation of theoretical and experimental data provides sophisticated information how nematocyst discharge processes could be used as nanomedical tools in cancer diagnostic and therapy. In this context the special consistence of the nematocyst cell wall is of highest interest. When testing various collagen hydrolysates in respect to their molecular composition it is instructive to carry out Quartz Crystal Microbalance (QCM) measurements [49] as complementary technique to NMR, AFM, SPR an MS [24,29,37,50]. QCM measurements allow the discrimination between triple helical and non-triple helical collagen fragments (Table 1). The results shown in Table 1 indicate that the triple helical collagen strands in mixtures of collagen fragments from jellyfishes exist and can be analyzed by QCM techniques. The raw material from cnidarian organisms (prepared according to Hoyer [51] and Sewing [52] degrade and dissociate above 45 °C in an irreversible way. It has to be emphasized that the source (e.g., fish or jellyfish) is not important for the occurrence of larger fragments but for the special production process. The upper section of Table 1 documents the results of measurements carried out over a total time period of 7000 s and a temperature limit up to 45 °C which is reached for the first time after 1500 s. When the temperature limit is 45 °C one can observe by the frequency alterations that the collagen strands are denatured and not reversible anymore. After 2500 s a low temperature region of 15 °C is reached again but the triple helical strands are denatured and did not renaturate anymore. A new increase of the temperature leads to a different profile of the Δν values. When altering the temperature between 15 °C and 37 °C only the frequency pattern, which is detectable, indicates a completely reversible processes in respect to the cnidarian collagen strands under study (as documented in the lower section of Table 1). The collagen strands remain triple-stranded and are proper target-structures for the proteoglycan saccharide chains as shown in Figure 5. Therefore, this method is beside NMR, SPR and AFM experiments in combination with molecular modelling calculations [24,36,37] a valuable supporting technique when nematocyst-like medical devices have to be improved.

### 2.4. Molecules Constituting the Nematocyst Membrane

To explain the nematocyst discharge process in a physically consistent way one has to consider the morphology and the constitution of the nematocyst cell membrane in which proteins and carbohydrates are interacting with each other in order to generate the elastic properties enabling the high pressure and velocity values during the Coulomb repulsion process. According to our theoretical model, this is much more important in the upper part of the nematocyst than in the lower part. Using this model description, morphological alterations of nematocysts which occur in Hydra, jellyfishes and sea-anemones can be handled using theoretically derived parameters in combination with experimentally obtained data. Triple-stranded collagen strands in the nematocyst cell wall which are associated with cysteine-rich mini-collagens interact with proteoglycans and nematogalectins and are therefore the building blocks for nematocyst-like nanomedical devices. Unspecific interactions between parts from the carbohydrate chains of proteoglycans with collagen strands (Figure 5) as well as highly specific interactions between certain moieties of proteoglycan carbohydrate chains with certain lectins (especially nematogalectins, [44,45,53]) (Figure 6a–c) have to be taken into account when the origin of the flexibility properties in the stylet-connected nematocyst’s tubule system is analyzed in molecular detail. Due to homology modelling, the nematogalectin structure could be constructed using the Swiss-model tool [54]: http://swissmodel.expasy.org/.

**Figure 7:**
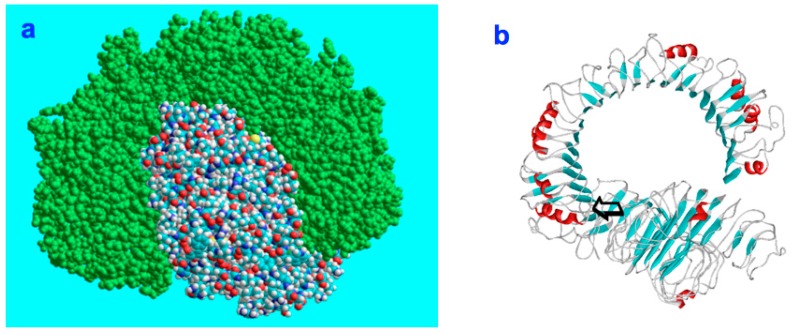
(**a**): CD14 (colored) and biglycan (green) in their space-filling states associate at a preferred contact region in order to establish a stable complex. (**b**): The initial contact point is marked by an arrow. The matrix component biglycan is proinflammatory and signals through Toll-like receptors 4 and 2 in macrophages [57,58,59].

**Figure 8:**
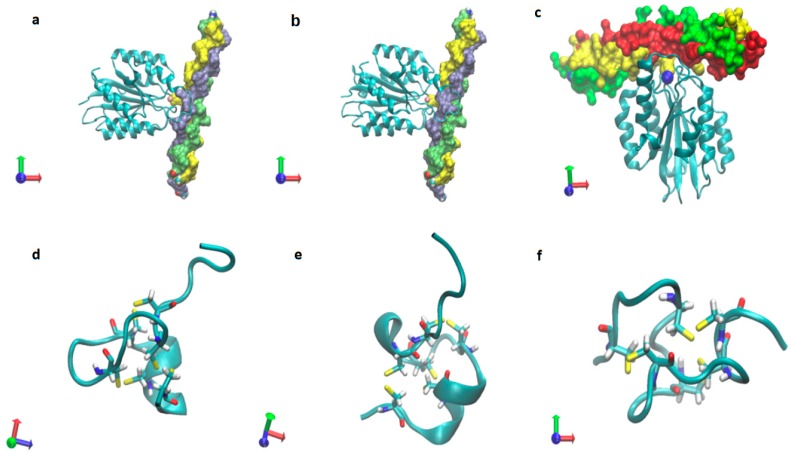
Triple-helical collagen in complex with integrins (**a**–**c**, in which a and b with pdb entery: 2m32.pdb, c with PDB entry: 1dzi.pdb, and mini-collagens (**d**–**f**, in which d with PDB entry: 1sop.pdb, c with PDB (Protein Data Bank) entry: 1sp7.pdb, f with PDB entry: 1zpx.pdb).

The rhamnose binding lectin [55] CSL3 (pdb-entry: 2ZX2.pdb) has been identified as the most suited template. The model structures are shown in Figure 6a–c. As described in the literature, the proteoglycan carbohydrate chains stabilize the cell-wall structure of the nematocyst in an essential way [56]. Model structures of the glycan and protein parts of proteoglycans are available [36]. Own molecular modelling calculations have shown that protein parts of proteoglycans, i.e., the leucine-rich biglycan [57,58,59] interact with other proteins in a highly specific way (Figure 7a,b). This is of importance when interactions with CD14 are in the focus [57,58,59], however, the interplay of such leucine rich molecules are also crucial players when the regeneration of the head region of Hydras is studied. In respect to the nematocyst membrane properties (Table 1) the glycan chains are of highest interest. Therefore, we have studied various proteoglycan fragments with molecular modelling methods concerning their interactions with collagen fragments and nematogalectins (Figure 5, Figure 6a–c, Figure 8a–f). All structural data sets concerning the key-molecules which constitute the nematocyst membrane and the wall of the stylet-connected tubule systems are considered in our molecular modelling studies. Molecular Dynamics (MD) simulations of collagen molecules [24] which are comparable to mini-collagen structures [60,61,62] in Cnidarian mini-collagens have a triple-helical core. This provides insights into the corresponding molecular organization processes in respect to their potential to interact with proteoglycans or collagen-binding proteins [23,36,37]. The corresponding interactions are mainly dominated by polar hydrogen bonds. Additional ionic interactions have to be considered. Unspecific associations of carbohydrate moieties from proteoglycans with collagen (Figure 5) as well as specific carbohydrate protein interactions mediated by nematogalectin (Figure 6a–c) were studied with molecular modelling method in the same way as described in the literature for receptor interactions with sialic acids and sulfated carbohydrate chains [24,25,27,38]. These data are used to test the reliability of the theoretical nematocyst model including all essential physical parameters. Our electron-microscopical (EM) analysis on nematocyst geometry has shown that the shape can be characterized as a combination of spherical and conical geometries (Figure 2). This is of importance for testing the predictions in relation to the theoretical five-stage model. Since the nematocyst discharge process is triggered by a mechanic-chemical signal which is initiated by sialic acid/polysialic acid molecules [63] on the skin of Cnidaria’s enemies and preys sialic acid coated nano-particles are extremely supportive artificial analytical tools beside naturally occurring sialic acid pattern on tumor cells [25,39]. In this context sialic acid conjugated Fluorescent Silica Nano Particles (FSNP-Sia) and functionalization of Semiconductor Quantum Dots (SQDs) with sialic acid are extremely helpful analytic tools (this will be outlined in a follow-up publication). Furthermore, additional NMR control-experiments have been designed. These control experiments were performed (this will also be discussed in a follow-up publication) using fish eggs [42] and analyzed by NMR with a similar setting as described for living eukaryotic cells [64]. The results of the one-dimensional NMR experiment will be shown in a follow-up publication because the first promising results suggest extended experimental series with various fish-eggs and nematocytes/nematocysts from different cidarian species. This experimental setting can be applied to nematocytes/nematocysts (Figure 1) but also to numerous tumor cells (Figure 2). Echinodermata, which are predators of Cnidaria, have developed sialic acids in evolution half a billion years ago. A number of special sialic acid molecules can be found in starfishes [65]. Therefore, it is instructive and intriguing to test various sialic acids as initiating molecules of the nematocyst discharge process. Our knowledge about the architecture of the nematocyst [66,67,68] including the consistence of its cell-wall and the stylet-connected tubule system allows us to discuss the question of in which way the presence or the absence of sulfate groups on the proteoglycan carbohydrate chains have any impact on the material characteristics. Although, in principle, sulfated proteoglycan fragments are able to interact with triple helical collagen fragments (Figure 5). We finally conclude that the absence or the presence of a sulfate group has a kind of sign-function because of the impact on the orbitals of the corresponding carbohydrate residue [36]. The knowledge concerning the evolution of Cnidaria and Echinodermata [69,70,71,72] enables us to discuss potential clinical applications in diagnostic and therapy under a nano-bionic aspect. Exploding nematocysts and their effect on tumor cells can be controlled by NMR as it has been shown for fish-eggs before and after their destruction. Artificial nematocyst-like devices could be used as powerful nanomedical tool which is able to attack polySia-rich tumor cells [43].

## 3. Discussions

In order to understand the nematocyst discharge process on a basic physical level, we combined approaches from applied and theoretical physics. When analyzing this process, a satisfactory model description allows deeper insights into basic physical details. Our theoretical model permits a discussion of the nematocyst discharge process in the dependence of key-parameters such as vessel morphologies, pressure values, forces and ejection velocities. With this model, we are able to depict the major effects leading to the stylet emission in an adequate way independent from structural deviations between the nematocysts of various species (Hydra, jellyfishes, box-jellyfishes and sea-anemones). The elastic properties of the materials surrounding the stylet near the operculum require a special molecular arrangement. It is necessary that the cell-wall sections in that region have a higher elasticity because they have to endure higher stresses than the rest of the cell membrane. Experimental data, including the parameters of various boundary conditions can be determined by a strategic combination of suited biophysical methods such as Surface Plasmon Resonance (SPR) [28], Atomic Force Microscopy (AFM) [24,37,48,73,74], nano-particle analysis supported by confocal laser scanning microscopy [75,76,77,78,79], Electron Microscopy (EM) (Figure 1 and Figure 2), NMR spectroscopy [37], Quartz Crystal Microbalance (QCM) techniques [49] (Table 1) and molecular modelling [25,36,38,39,40,41] (Figure 5, Figure 6, Figure 7 and Figure 8) provide essential biophysical data about the morphological and material characteristics of nematocysts. It is therefore possible to use these data as a first concept in a feasibility study for the construction of nematocyst-like nanomedical diagnostic devices, i.e., tailor-made nematocysts which contain therapeutic drugs. These nanomedical devices have to be effective and specific in respect to various carbohydrate signatures in the glycocalyx of tumor-cells. For this purpose, it is essential to understand the target-directed nematocyst discharge process in all aspects of the fundamental physical laws. The biochemical properties which are correlated with these laws can be modified accordingly when nematocyst-like nanomedical devices are constructed. 

### Physical Properties of Nematocysts and Their Potential Targets

In non-equlibrium thermodynamics [80] one has to describe the intact nematocyst in its metastable state first. This state will be reached after a contact of the intact nematocyte with sialic acids. The mechanic-chemical contact to the sialic acid sensitive receptor is therefore responsible for non-reproducible fluctuations. If the stationary state of the process is metastable, then non-reproducible fluctuations involve local transient decreases of entropy. The reproducible response of the system increases the entropy back to its maximum by irreversible processes: the fluctuation cannot be reproduced with a significant level of probability. Fluctuations about stable stationary states are extremely small except near critical points [80]. The information which we derived by methods from applied and theoretical physics are sufficient to describe the complete nematocyst discharge process in its five stages. Furthermore, the initial release mechanism triggered by an interaction of cell-surface exposed receptor with sialic acid molecules can be studied in detail using biophysical methods in combination with suited model systems [25,38,41,42]. NMR experiments, molecular dynamics (MD) simulations and microscopic studies provide complementary information to our theoretical five-stage model description. These results are the basis for a follow-up NMR study on fish-eggs, nematocysts, tumor cells and sialic acid coated nanoparticles [75,76,77] which are a prerequisite for the corresponding clinical studies. Cancer cells have distinct morphological parameters and a special signature of carbohydrate chains on their cell surface in which sialic acids play a prominent role. Polysialic acid (building block: Neu5Ac-alpha2-8-Neu5Ac) in high concentration on the surface of cells (e.g., fish eggs [42] or tumor cells [25,38,43] can initiate the nematocyst discharge process. However, in some cases, it is necessary to increase the sialic acid concentration in the vicinity of the nematocysts to support the discharge processes. It is an advantage for our strategy that the physical properties of cancer cells in respect to their plasticity differ in a significant way from normal cells as it turned out after TEM (transmission electron microscopy) studies on neuroblastoma (Figure 2I,II) but also on glioblastoma (own observation) cells. The cancer cells under study are much weaker and show a higher degree of protrusions than normal tissue cells. These findings are of importance since neuroblastoma and glioblastoma cells which carry the polySia molecules on their surfaces are suited targets for modified nematocysts which are able to attack cancer cells in a specific way. Our five-stage theoretical model which describes the different phases of the nematocyst discharge process is a first step aiming in a complete adaption of such a concept for diagnostic and therapeutic applications which focus on a target-directed destruction of cancer cells. 

With the five-stage model, we can explain the basic physical properties of the nematocyst discharge process providing the force and the energy for the ejection of the toxic stylets when Cnidaria are defending the attacks from their predators. Molecular dynamics (MD) simulations of collagen fragments [24] and of crucial parts of the proteoglycan carbohydrate chains [36] (Figure 5) which are forming the structures of nematocyst cell membrane as well as electron microscopical studies on the shape variations of nematocysts (Figure 1) deliver the boundary conditions for the five-stage model, such as elasticity, pressure and other material-dependent parameters, e.g., Figure 4 and Table 1. Values for the conical and spherical geometries of the nematocyst vessels and the shape variations of their stylets (Figure 1 and Figure 3) are also of importance. The number of the nematocyst discharge processes strongly depends on the concentration of polySia molecules in the environment of the cells (own observation) and will be analyzed in more detail in a follow-up study with labeled neuraminic acid molecules [75,76,77,78,79]. The chain lengths of polySia seem to play only a minor role (Figure 5) as revealed by microscopy and NMR spectroscopy [38,48]. The spider lectin SHL-1 [23] other small lectin-like structures have to be considered as proper polySia interacting peptides [38,39,48] on nematocyst-like nanomedical devices. The amino acid sequence of the effector domain of the myristoylated alanine-rich kinase C substrate (MARCKS-ED) is such a candidate. The amino acid sequence indicates by its composition that arginine and aromatic amino acid residues play a crucial role for a specific polySia binding. This is also the case for the spider lectin SHL-1 [38,39]. In particular, the role of aromatic amino acid residues is underlined by amino acid sequence of the MARCKS-ED control peptide [38] which has no binding specificity for polySia. The phenylalanines that were changed to alanines in the control peptide support polySia binding of MARCKS-ED but the alanine residues in the control peptide are not. As a first test for the effect of exploding nematocysts suited cells are necessary. In our case we have chosen fish eggs which have polySia in high concentration on their surface in a similar way than various tumor cells [42,43]. The state of the fish egg before and after its destruction can be documented by 1D NMR spectra (Figure 2II and Supplementary Materials). Besides microscopic studies, it is therefore also possible with NMR experiments [64] to figure out in which way an increased concentration of sialic acids and polySia fragments are able to support the initiation of the nematocyst discharge process. Of special interest are polySia glycomimetics, e.g., Tegaserod [38,81,82], which are also interacting partners of sialic acid receptors. It has to be emphasized that the polysaccharide polySia in which the sialic acid Neu5Ac building-blocks are linked by an α2-8 bond to each other are the natural triggers which are responsible for the inition of a nematocyst discharge event. Beside sialic acid binding other specific carbohydrate—protein interactions play a crucial role in this study and concern nematogalectins. Galectins [83,84,85,86,87] are galactose binding animal lectins which have various biological functions. In respect to nematogalectins, it is of importance to clearly identify the corresponding contact region of this cnidarian lectin (Figure 6a–c). Also, in this case, the benefit of molecular modelling *in silico* calculations under special consideration of *ab initio* calculations is obvious [24,25,36,38,39,40,41,88,89], since this theoretical approach shows under which prerequisites nematocyst-like nanomedical devices can be constructed; in detail: which material requirements are necessary [90,91,92,93,94,95,96,97,98,99,100]. Fast-growing tumor cells typically have more permeable membranes than healthy cells, allowing the leakage of marked sialic acid molecules into their cell body. Moreover, tumor cells lack an effective lymphatic drainage system, which leads to subsequent accumulation of these particles. Since sialic acids (especially Neu5Ac) initiate the nematocyst discharge process labeled Neu5Ac molecules, even free in solution could be used to trigger the nematocyst discharge process and will also be helpful markers inside the nematocyst. Therefore, sialic acid coated nanoparticles are suited tools to improve the efficiency of our approach [75,76,77,78,79]. Besides a target-directed attack against tumor cells, nano-bionic nematocyst-like devices from cnidarian origin could in principle be applied for therapies which support neuronal regeneration because the state of neuronal de- and re-differentiation depends also on the polySia concentration on the nerve cell surfaces ([25,38] and literature cited within). Additionally, in the case of treatments against certain pathogens (viruses and bacteria) [39,40,41] the use of sialic acid sensitive nematocyst-like vesicles has to be considered as promising diagnostic and therapeutic option, especially, when multiresistent pathogens have to be taken into account. Our approach in which nematocysts are used as bionic blueprints has therefore multiple benefits in the fields of nanomedicine and nanopharmacology. Especially, the theoretical model described here enables us to modify the nematocyst architecture and adapt it for diagnostic and therapeutic clinical applications. It is essential in this context to understand the role of nematogalectin as a component of the tubule system and its interactions with mini-collagen strands and proteoglycans. As outlined here, corresponding experiments on triple helical collagen fragments can be performed by a combination of QCM techniques (Table 1) and molecular modelling methods (Figure 5, Figure 6a–d). These results have to be combined with NMR data from cellular test-systems. Such suited cellular test systems are fish-eggs which carry polySia on their cell surfaces. The high tumbling rate of the surface exposed bio-macromolecules lead to broad signals of the one-dimensional proton NMR spectrum (not shown). After the destruction of the fish eggs, highly resolved signals from the inside of the cell can be detected. This is also the case when sialic acid coated nanoparticles are studied.

## 4. Materials and Methods 

### 4.1. Tumor Cells: Neuroblastoma Cells

Cell line SK-N-AS corresponding to neural bone marrow metastasis cells from a human source were purchased from American Type Culture Collection (Manassas, VA, USA). Glioblastoma cells: Cells were taken from a glioblastoma multiforme (WHO stage four).

### 4.2. Hydra Culture

Experiments were carried out using *Hydra vulgaris* strain AEP. Animals were cultured according to standard procedures [101].

### 4.3. Proteoglycans, Algae Polysaccharides and Collagen Fragments from Marine Organisms and Bacteria

Fish cartilage [102], green algae [39,103] and Cnidaria (*Rhopilema esculentum*) [36,37,51,52] are our sources for proteoglycans, polysaccharides and collagen-fragments. Polysialic acid (polySia): Polisialic acid fragments of various chain lengths are derived from colominic acid of *E. coli*.

### 4.4. Electron Microscopy

Specimen were fixed in 6% glutaraldehyde in 0.1 M Na-cacodylate buffer overnight, rinsed three times for 10 min in 0.1 m Na-cacodylate buffer, and postfixed in 1% osmiumtetroxide (OsO4) for one hour. Samples were dehydrated in a series of alcohol (50%, 70%, 80%, and 96% ethanol for 15 min, 100% ethanol for 1 h), and embedded in Epon. Semithin sections (1 µm) were stained with methylene blue. Ultrathin sections (100 Å) were contrasted with lead citrate (2%) and uranylacetate (0.5%) and examined in a Zeiss 109 electron microscope. 

### 4.5. Measurements with a QCM System

The quartz crystal microbalance system QCM 200 SRS (Stanford Research Systems, 1290-D Reamwood Ave, Sunnyvale, CA 94089, USA) was used to analyze jellyfish collagen and collagen hydrolysate samples under various temperature conditions. 

### 4.6. Molecular Modelling

The conformational analysis and energy optimization of sulfated and non-sulfated glycans was carried out with Hyperchem 8.0 Prof. (using the CHARMM27 force field, Chemistry at HARvard Macromolecular Mechanics, Cambridge (Massachusetts) USA) and Gaussian16 (B3LYP/6-31G*). Glycan-protein docking was performed with the Molegro 5.0 trial version. For protein-protein docking calculations the Webserver Haddock 2.2 version was used. MD-simulations were carried out with YASARA v.19.4.29 using the YASARA force field under physiological conditions [36,104,105,106] and additional references in Supplementary Materials.

### 4.7. Preparation of FSNP-Sia

FSNP-Sia was synthesized using fluorescein-doped silica nanoparticles (FSNP) and the photocoupling strategy to conjugate Sia on FSNP, following previously developed protocols [75,76,77,78,79,107]. 

#### 4.7.1. Synthesis of Fluorescent Silica Nanoparticles (FSNP)

Fluorescein 5-isothiocyanate (39 mg) was dissolved in anhydrous ethanol (16 mL) and (3-aminopropyl) trimethoxysilane (17 µL) was added while stirring. The mixture was stirred overnight at 42 °C to produce the precursor solution. For the synthesis of fluorescein isothiocynate (FITC)-doped silica nanoparticles, 5.0 mL of the precursor solution was added to anhydrous ethanol (34 mL) while stirring followed by the addition of tetraethyl orthosilicate (1.7 mL) and aqueous ammonia (1.4 mL, 25% v/v). The mixture was stirred for 48 hours to give the FSNP solution.

#### 4.7.2. Synthesis of Perfluorophenyl Azide (PFPA)-functionalized Fluorescent Silica Nanoparticles (FSNP-PFPA)

FSNP-PFPA was synthesized by adding a solution of silane-derivatized PFPA in toluene (7.0 mL, 10 mg/mL) into the FSNP solution and stirring at room temperature for 24 h. The product was purified by repeated washing and centrifugation in acetone (12,000 rpm, 30 min), and was re-suspended in 10 mL of acetone to give FSNP-PFPA. 

#### 4.7.3. Synthesis of Sialic Acid-Conjugated FSNP (FSNP-Sia)

To 1 mL of FSNP-PFPA in acetone, an aqueous solution of sialic acid (1.0 mL, 3.6 mg/mL) was added. The glass jar containing the mixture was covered with a 280 nm long-path optical filter and was subsequently irradiated with a 450 W medium-pressure mercury lamp for 40 minutes. The resulting FSNP-Sia was purified by repeated washing in Milli-Q water and centrifugation (12000 rpm, 30 min). The final product was re-suspended in water. 

## 5. Conclusions

The nematocyst discharge process is initiated by a mechanic-chemical interaction when an object is in contact with the surface of a nematocyte carrying an intact nematocyst. Furthermore, sialic acids or polySia molecules have to be involved. Data about the molecular details of this procedure obtained from theoretical approaches in combination experimentally derived results enables us to consider the possibilities of nematocysts as blueprints for nanomedical devices in tumor cell diagnostic and cancer therapy. The chemical reactions related to the nematocyst discharge mechanisms mainly depend on the de-protonation and the swelling process of γ-polyglutamate. In the case of a nematocyst discharge, which is caused by sialic acids, the chemical and physical-mechanic data can be separated [1]. As described in our five-stage model, one has to take into account that the sialic acids do not occur in Cnidaria. Using our theoretical model, it is possible to consider nematocysts of various species with different morphologies for further studies. Sialic acid molecules which play also a crucial role in developmental processes [90,91] were invented during the evolution in Echinodermata (e.g., starfishes). Therefore, Cnidaria (e.g., Hydra, sea-anemones, jellyfishes) have developed a strategy to protect themselves against the attacks of these enemies, at first Echinodermata, by a diffusion-dependent polySia sensitive defense system (related to the concentration of sialic acid molecules and their density on the cell-surfaces). The monosaccharide sialic acid which forms the alpha2-8 linked polysialic acid (polySia) has been identified as key-molecule which identifies a predator. A potential application in nanomedicine is possible since polySia occurs also in high concentration in the glycocalyx of tumor cells and are responsible for their degree of malignancy. When the experimental methods applied here are supported by approaches from theoretical physics it is feasible to invent new diagnostic and therapeutic concepts flanked by encapsulation and target strategies. In detail, similar cell surface signatures which induce the nematocyst discharges are also specific target structures on highly malignant cells [92,93]. These carbohydrate target structures undergo specific interactions with lectins [22,38,78,94] but possibly also with carbohydrate chains [95,96,97]. Besides encapsulation and targeting of potent anti-tumor drugs, its identification [98,99,100] also plays a crucial role. Polysaccharides from marine organisms [39,102,103] provide homogenous encapsulation materials. In combination with specially prepared collagen fragments [29,50] it is possible to construct nano-bionic medical devices [107,108,109,110] for which nematocysts are the blueprints. In summary, our investigated theoretical model describes a nematocyst discharge process which is initiated by a contact with sialic acids [111] causing a Coulomb repulsion [112] which leads to the ejection of a toxic stylet [113]. This nano-bionic model has inspired us to analyze such processes under various boundary conditions aiming at new diagnostic and therapeutic approaches with innovative nanomedical devices. Besides the shape of the nematocyst, the consistence of the nematocyst membrane and the stylet-connected tubule system are important to figure out how the sialic acid dependent triggering of the nematocyst discharge process in an intact nematocyte can be used for cellular targeting. Our theoretical and experimental approaches enable us to decide which are the most promising nanomedical blueprints for our endeavor to establish a target-directed tumor cell attack, e.g., in the field of neuro-oncology. Since highly malignant brain tumor cells are coated on their surfaces with polysialic acid (polySia) molecules similar like fish-eggs we use these insights for a strike against the Achilles heel of cancer cells. Therefore, we have to identify the most effective toxin which is able to block the growth and the spreading of brain tumor cells such as glioblastomas. These toxins have to be encapsulated in a nanomatrix which consists of liposomes/micelles, polysaccharide- and collagen-fragments (especially mini-collagens, Figure 8d–f) in a similar composition as found in nematocysts. The target-directed intervention of the toxin-loaded nanoparticles in the dependence of the polySia rich contact structures on the brain tumor cells is performed by polySia binding mini-lectins on the surfaces of nematocyst-like nanoparticles.

## Figures and Tables

**Table 1:** Measurements on triple and non-triple helical collagen fragments from exumbrellatissue from the jellyfish species *Rhopilema esculentum* [36,37,51,52] have been performed on the Quartz Crystal Microbalance system QCM 200 SRS. The differences of the detected frequencies are given by Δν in Hz. The experimental temperature is T in °C and the experimental time is t in s. In the table on the top 45 °C has been chosen as highest temperature which leads to a denaturation of the collagen strands. In the case 37 °C is the highest temperature (table on the bottom) no denaturation of the same material is detected.

**Δν, Hz**	**0**	**300**	**250**	**300**	**−300**	**300**	**0**	**−300**	**−100**	**150**
T, °C	15	30	45	40	15	45	30	15	15	15
t, sec	>0	>1000	>1500	>2000	>2500	>4000	>4500	>5000	>5500	>6000
**Δν, Hz**	**0**	**700**	**300**	**100**	**700**	**300**	**100**	**700**	**300**	**100**
T, °C	15	37	25	15	37	25	15	37	25	15
t, sec	0	1500	2000	2500	3500	4000	4500	6000	6500	7000

## Supplementary Materials

The following are available online at https://www.mdpi.com/1660-3397/17/8/469/s1, Comparison between a 1D proton NMR spectrum of intact fish-eggs with a 1D proton-NMR spectrum of the same fish-eggs which are destroyed in a nematocyst discharge event.
