# Supplementary material for: The Sialic Acid-Dependent Nematocyst Discharge Process in Relation to Its Physical-Chemical Properties Is a Role Model for Nanomedical Diagnostic and Therapeutic Tools
Source: Mar Drugs. 2019 Aug 12;17(8):469. doi: 10.3390/md17080469 (PMC6722915; doi:10.3390/md17080469)
Supplement: Supplementary file 1 [file marinedrugs-17-00469-s001.pdf]

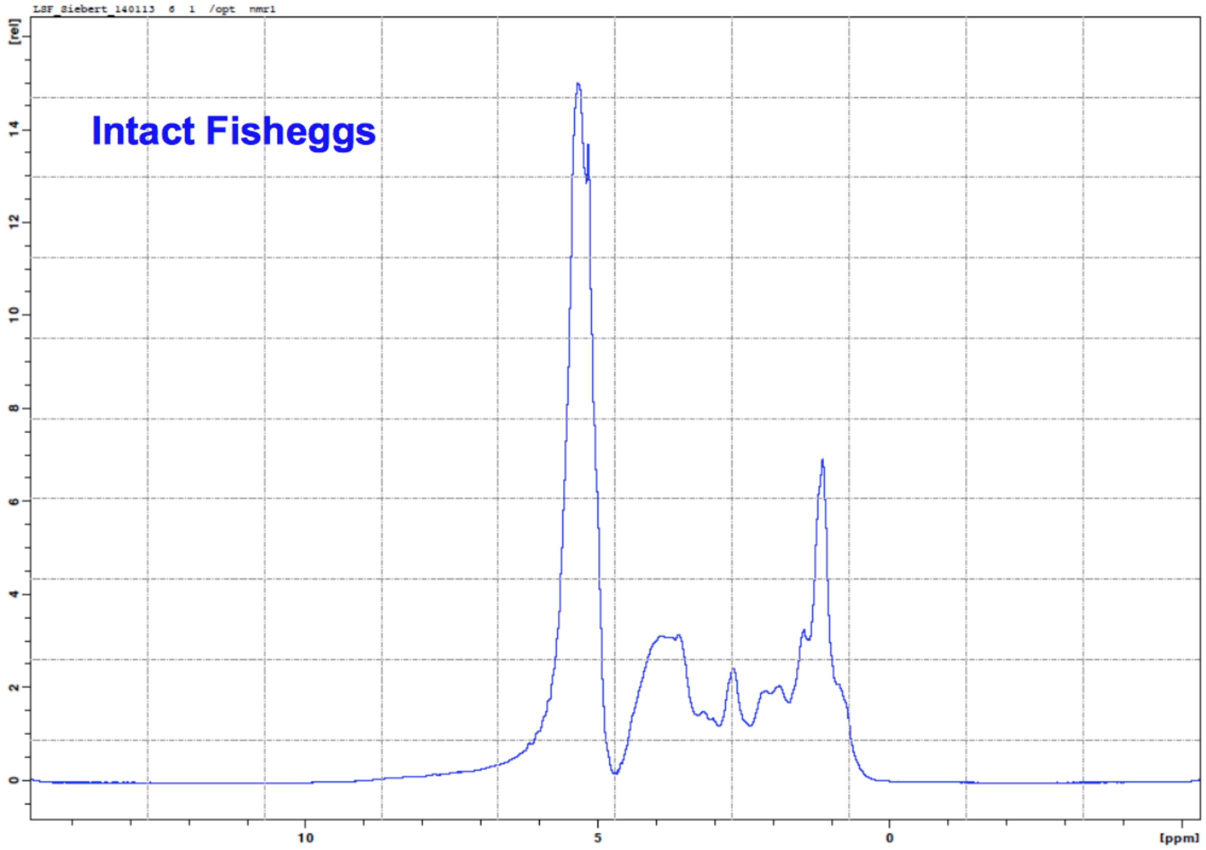
LSF_Siebert_140113 6 1 /opt nmr1
Intact Fisheggs
[rel]
14
12
10
8
6
4
2
0
10
5
0
[ppm]

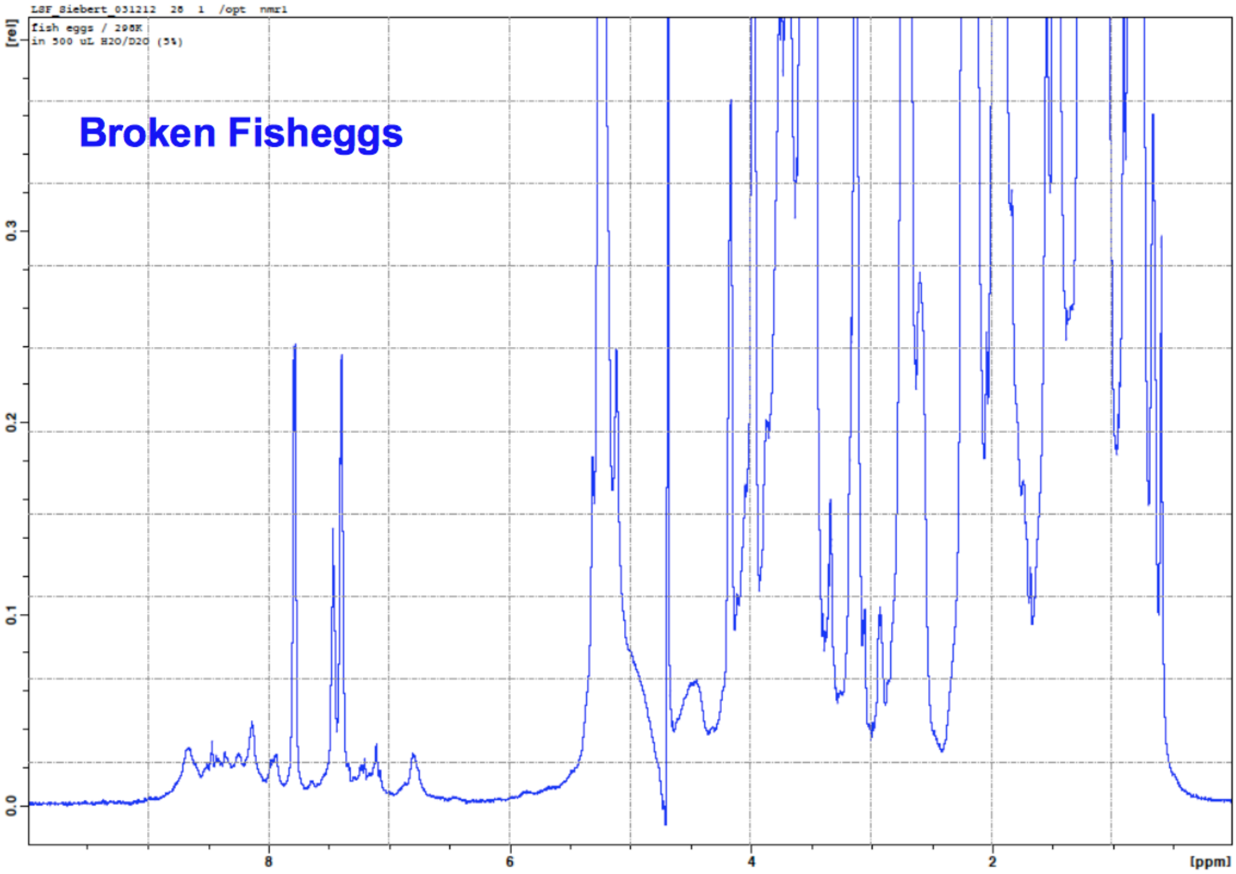
LSF_Siebert_031212 26 1 /opt nmr1
fish eggs / 298K
in 500 uL H2O/D2O (5%)
Broken Fisheggs
[rel]
0.3
0.2
0.1
0.0
8
6
4
2
[ppm]

Comparison between a 1D proton NMR spectrum of intact fish-eggs with a 1D proton-NMR spectrum of the same fish-eggs which are destroyed in a nematocyst discharge event.

Additional references:

Hyperchem 8.0.6, Molecular Visualization and Simulation Program Package (Hypercube Inc, Gainesville, **2007**)

Gaussian 16, Revision B.01, Frisch, M.J., Trucks, G.W., Schlegel, H.B., Scuseria, G.E., Robb, M.A., Cheeseman, J.R.; Scalmani, G.; Barone, V.; Petersson, G.A.; Nakatsuji, H.; Li, X.; Caricato, M.; Marenich, A.V.; Bloino, J., Janesko, B.G., Gomperts, R., Mennucci, B., Hratchian, H.P., Ortiz, J.V., Izmaylov, A.F., Sonnenberg, J.L., Williams-Young, D., Ding, F., Lipparini, F., Egidi, F., Goings, J., Peng, B., Petrone, A., Henderson, T., Ranasinghe, D., Zakrzewski, V.G., Gao, J., Rega, N., Zheng, G., Liang, W., Hada, M., Ehara, M., Toyota, K., Fukuda, R., Hasegawa, J., Ishida, M., Nakajima, T., Honda, Y., Kitao, O., Nakai, H., Vreven, T., Throssell, K., Montgomery Jr., J.A., Peralta, J.E., Ogliaro, F., Bearpark, M.J., Heyd, J.J., Brothers, E.N., Kudin, K.N., Staroverov, V.N., Keith, T.A., Kobayashi, R., Normand, J., Raghavachari, K., Rendell, A.P., Burant, J.C., Iyengar, S.S., Tomasi, J., Cossi, M., Millam, J.M., Klene, M., Adamo, C., Cammi, R., Ochterski, J.W., Martin, R.L., Morokuma, K., Farkas, O., Foresman, J.B., Fox, D.J. *Gaussian, Inc., Wallingford CT* **(2016)** GaussView 5.0. Wallingford, E.U.A.
